# Investigation on *Brucella* infection in farm animals in Saham, Sultanate of Oman with reference to human brucellosis outbreak

**DOI:** 10.1186/s12917-019-2093-4

**Published:** 2019-10-28

**Authors:** Yasmin ElTahir, Anfal Al-Farsi, Waleed Al-Marzooqi, Alghalya Al-Toobi, Osman M. Gaafar, Maryne Jay, Yannick Corde, Shekar Bose, Abeer Al-Hamrashdi, Kaadhia Al-Kharousi, Sunil Rajamony, Muhammed Nadeem Asi, Nasseb Al-Saqri, Rudaina AlBusaidi, Elshafie I Elshafie, Eugene H. Johnson

**Affiliations:** 1 Department of Animal & Veterinary Sciences, College of Agricultural & Marine Sciences, Sultan Qaboos University, P.O.box 34, 123 Alkhod, Sultanate of Oman; 2 Paris-Est University/Anses, EU/OIE/FAO & National Reference Laboratory for Brucellosis, Animal Health Laboratory, Maisons-Alfort, France; 3 Department of Natural Resources Economics, Sultan Qaboos University, P.O.box 34, 123 Alkhod, Sultanate of Oman

**Keywords:** Brucellosis, *Brucella melitensis*, Saham, Oman

## Abstract

**Background:**

The objective of this study was to investigate *Brucella* infection in farm animals in Saham, Oman, with reference to a survey carried out by the Ministry of Agriculture & Fisheries (MAF) for Brucellosis during the period of May to July 2016 in Saham, following an outbreak of human brucellosis. We wanted to apply different serological, bacteriological and molecular tests in a time frame (phase 1, 2 & 3) with reference to the pivotal time of a human brucellosis outbreak to ascertain the status of the disease in Saham area where the MAF survey was conducted. Blood samples were collected from farm animals and sera were screened in parallel for *Brucella* antibodies using different serological tests.

**Results:**

Using the RBT test, phase 1 sera showed seropositivity in sheep at 2.6%, (95% CI: 0.5–13.5%), in camel (5.9%, 1.1–27.0%), but not in sera from goats and cattle (0%). Using I-ELISA, seropositivity in goat was 3.1% (0.6–15.8%), with no positive sheep and cattle. Using c-ELISA for camel we found a seropositivity of 5.9% (1.1–27.0%). Furthermore, CFT seropositivity in goats was 21.9% (CI: 11.3–38.9), cattle and sheep sera were negative and camel was 5.9% (1.1–27.0%).

In phase 2, the seropositivity in goats was 1.9% (1.4–2.6%), sheep 4.5% (3.5–5.8%), cattle 1.1%, (0.5–2.3%) and camels 18.2% (5.1–47.7%),

Phase 3 sera were collected 6 months after the human brucellosis outbreak. With RBT, the seropositivity in goats was 3% (1.0–8.5%), sheep 2% (0.6–7.1%) cattle 1% (0.2–5.5%). With I-ELISA, goats & camels were negative, sheep were 3% (1.0–8.5%) and cattle 1% (0.2–5.5%).

Moreover, *B. melitensis* was isolated from a bronchial lymph node of the RBT and I-ELISA seropositive cow and confirmed by Multiplex PCR and biochemical tests.

**Conclusion:**

Using a retrospective study analysis of animal sera and following up after a human brucellosis outbreak, the present study showed a slight decrease in seropositivity of infected animals after the MAF implemented test and slaughter policy. The most interesting finding in this study was the isolation, identification and molecular characterization of *Brucella melitensis* in a cow (spillover), which is not a preferential host for *Brucella melitensis*.

## Background

Brucellosis is one of the world’s most widespread zoonotic diseases affecting both, public health and animal production [1–3] The disease is widely distributed, particularly in the Middle East and is endemic in the southern region of Oman, where the annual incidence exceeds a thousand cases per million [4], with the highest zoonotic impact being attributed to *Brucella melitensis* followed by *B. abortus* and *B. suis*. According to the Office for International des Épizooties (OIE), the disease is also classified as one of the neglected zoonosis with a serious animal and public health importance throughout the world [5, 6].

A major impact of the disease is on livestock productivity resulting in major losses for international trade [7] as well as cash income [8]. Added to that is the impact on sustainability of farming resulting from abortions and reduced milk production as a consequence of the disease and its complications.

Infection is transmitted from infected animals through colostrum, milk or uterine discharges following abortion or parturition [9] in which reside actively infective bacteria. Humans become infected with *Brucella* through the ingestion of raw milk and other dairy products or by direct contact with contaminated tissue, blood, urine, vaginal discharges, aborted fetuses and placentas [10].

In Oman, the focus is on the livestock sector as part of a strategy to diversify its economy as this sector is playing a crucial role in provision of and employment for the population to food security.

Nonetheless, livestock production is under continuous threat by existing and emerging diseases that may result in direct and indirect losses to the livestock owner as well as to the national economy [11].

One of the threats and under-researched livestock diseases in Oman is brucellosis. Human cases are mainly restricted to the Dhofar Governorate since the human brucellosis surveillance program began in 1991. The rest of the governorates of Oman has shown a consistently low incidence of the disease, but a marginal increase has been observed in recent years, peaking during the year 2016 [12].

Between May and November 2016, the Ministry of Health reported 75 confirmed human cases of brucellosis in Saham in AlBatinah governate in the Sultanate of Oman. Several patients had a history of consuming a locally produced goat cheese. The local cheese producer had over 100 goats and a cow in his farm, which was his traditional family business. The Ministry of Agriculture and Fisheries (MAF) found seropositive animals herds represented by 43 out of 2211 goats (1.9, 95% CI: 1.4–2.6%), 55 sheep out of 1230 (4.47%, 3.5–5.8%) 6 cattle out of 565 (1.06%: 0.5–2.3%) and two out of 11 camel (18.18%, 5.1–47.7%) when tested in parallel with both RBT and I-ELISA or C-ELISA tests. The increase in human brucellosis cases called for action by MAF which adopted policies including, tightening quarantine measures and slaughtering susceptible animals.

The aim of the present study was to investigate the serological profile of *Brucella* infection in animals in Saham before, during and after the human brucellosis outbreak. Cultural and molecular techniques were also applied to identify the *Brucella* species involved in the infection and to assess the control measures taken by MAF in Saham [13].

## Results

Serum samples collected from goats, sheep, cattle and camels were analyzed serologically for detection of Brucella antibodies using RBT, ELISA and CFT at different phases of the study (phase 1, 2 & 3).

Details form the testing using of sera from Phase 1 using the different methods are shown in Table 1. As presented in the table, no sheep or cattle were seropositive, while results differed between tests for goats and camels.

**Table 1:** Seroprevalence of *Brucella* infection in goats, sheep, cattle and camels in Saham using individual serological tests (RBT, ELISA and CFT) before the human outbreak

Animal species	No.	Male	Female	Number of positive samples by Serological tests (prevalence%, 95 confidence interval					
				RBT	I- or C-ELISA	CFT	RBT/ELISA	RBT/CFT	RBT/CFT/ ELISA
goat	32	12	20	0	1 (3.1, 0.6–15.8%)	7 (21.9, 11–38.8%)	0	1 (3.1, 0.6–15.8%)	0
sheep	38	15	23	1 (2.6, 0.5–13.5%)	0	0	0	0	0
Cattle	1	0	1	0	0	0	0	0	0
camel	17	7	10	1 (5.9, 1.1–27%)	1 (5.9, 1.1–27%)	1 (5.9, 1.1–27%)	1 (5.9, 1.1–27%)	1 (5.9, 1.1–27%)	1 (5.9, 1.1–27%)

*RBT* Rose Bengal Test, *ELISA* indirect Enzyme-linked Immunosorbent Assay, *CFT* Complement Fixation Test, *N* No abortion History, *NN* unknown abortion history

Results of testing of sera from **Phase 2** is shown in Table 2. Seropositive animals (RBT& ELISA) were found for all animals, but highest for camel (18.2%) followed by sheep (4.5%) goat (1.9%) and cattle (1.1%) No details about the animals tested were available.

**Table 2:** Seroprevalence of *Brucella* infection in goats, sheep, cattle and camels in Saham using individual serological tests (RBT, ELISA) during the human Brucellosis outbreak

Animal Species	Collected samples	Number of positive samples by Serological tests (RBT & ELISA) (prevalence%, 95 confidence interval)
Goat	2211	43 (1.9%, CI: 1.4–2.6%)
Sheep	1230	55 (4.5%, CI: 3.5–5.8%)
Cattle	565	6 (1.1%, CI: 0.5–2.3%)
Camel	11	2 (18.2%, CI: 5.1–47.7%)

The results of testing of sera from **Phase 3** are shown in Tables 3 & 4. No seropositive camels were found, an only low numbers of goat, sheep and cattle, a bit varying with different methods.

**Table 3:** Seroprevalence of *Brucella* infection in livestock in Saham (phase 3) using serological tests (RBT, I-ELISA)

Animal species	No.	Male	female	Number of positive samples by Serological tests (prevalence%, 95 confidence interval)		
				RBT	I-ELISA	RBT/I-ELISA
Goat	100	31	69	3 (3, 1–8.5%)	0	0
Sheep	99	47	53	2 (2, 0.6–7.1%)	3 (3, 1–8.5%)	1 (1, 0.2–5.5%)
Cattle	99	25	74	1 (1, 0.2–5.5%)	1 (1, 0.2–5.5%)	1 (1, 0.2–5.5%)
Camel	101	20	81	0	0	0

**Table 4:** Summary of Phase 3 seropositive goats, sheep, cattle and camels in Saham (phase 3) using serological tests (RBT, I-ELISA or c-ELISA for camel sera )

Species	RBT		ELISA	
	Positive / Tested	Prevalence % (95% CI)	Positive / Tested	Prevalence% (95% CI)
Goats	3/100	3%	0/100	0%
Sheep	2/99^a^	2.02%	3/99^a^	3.03%
Cattle	1/99^b^	1.01%	1/99^b^	1.01%
Camels	0/101	0%	0/101	0%

^a^(1/3) sheep was positive in two tests.

^b^The same animal was positive in two tests.

### Identification of *Brucella* from bacterial isolate

**Fig. 1:**
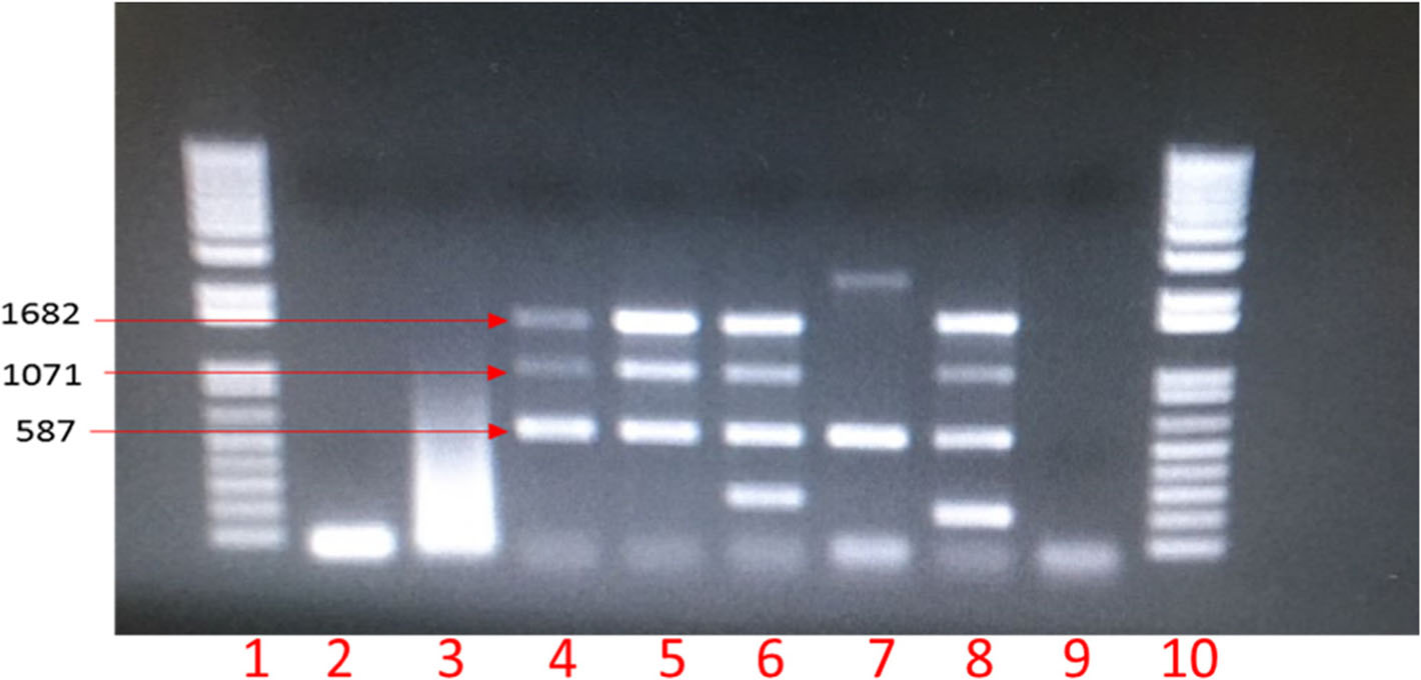
Agarose gel electrophoresis of species-specific Multiplex PCR products. Lane 1 and 10 are1 kb plus ladder. Lane 2 and 3 are negative *Brucella*-negative samples. Lane 4 is the original bacterial isolate. Lane 5 contains the subculture. Lane 6, 7, 8 are positive controls (*Brucella* suis, RB51 and Rev. 1 respectively) and 9 contains a negative PCR control

Twenty five samples including bronchial lymph nodes, spleen and mesenteric lymph nodes were collected from a serologically positive cow. A positive isolate was obtained from bronchial lymph nodes but not from the other tissues though they were positive by immunohistochemistry (data not shown). This could be due to degenerated microorganisms or a deficient isolation technique [14]. As shown in Fig. 1, the multiplex PCR using DNA from bacterial isolate from the bronchial lymph node showed three distinct bands of sizes 1682, 1071 and 587 bp, characteristic of *B. melitensis.*

## Discussion

In the light of overall results (Tables 2 & 3) we conclude that there is clear tendency towards a decrease in the seroprevalence of brucellosis in the 6 months after the human outbreak. This was most expressed in camels, from 18.2% (5.1–47.7%) to 0%, and sheep from 4.5% (3.5–5.8%) to 1% (0.2–5.5%) in Saham. In goats, a few (*n* = 3) tested positive in RBT 6 months after, but the ELISA test was negative. The seroprevalence in cattle remained the same (1%). Interpretation is restricted here, due to the low number of reactors after the outbreak.

A critical observation on animals sampled was the lack of management and hygiene reflected in improper placement plan where new animals were introduced without quarantine or serological analysis. Added to that was the dispatch of aborting animals and indiscriminate placement of animals irrespective of infection status, age or species. More to that was the low hygiene where contamination of feed and water was rampant given the improper disposal of aborted materials and improper animal husbandry. In this study, the owner of the seropositive cow reported that he introduced a new bull to his farm, from an endemic area in Dhofar Governate and the bull was not tested for *Brucella* antibodies. This information led us to suspect that the bull was the source of the infection. Unfortunately, the owner refused when we offered to test the bull to rule out the source of the infection.

In this study (Tables 1, 3), a number of sera tested positive in one test only RBT, CFT or I-ELISA which might lead to inaccurate statistics in any epidemiological study. We have presented all results, and our data do not support any method comparison study. Some serum samples which were found as positive by RBT were found negative by I-ELISA. This might result from cross-reacting antibodies in the RBT. RBT has been widely and successfully used for screening in brucellosis control programs. However, Diaz-Aparicio et al. [15] reported that RBT failed to detect a number of infected goats by the standard procedure recommended for the test, but when they modified the antigen-serum ratio to 1:3 they obtained 100% seropositivity, where in our study the ratio was 1:1. Thus further investigation is warranted to determine whether it is the case with goats, cattle and camels in Saham area.

Several researchers reported that I-ELISA is more sensitive than conventional serological tests. Discrepancies between the two tests in the present study could be due to several reasons, including differences in sensitivity and specificity as I-ELISA was reported to be more sensitive than RBT [16] and RBT more specific than the I-ELISA [17]. In phase 2 of this study, MAF indicated that seropositive animals were the ones tested positive in both RBT & ELISA. There was no detailed information of the performance of each test alone or the combination of the two tests together for the rest of the animals.

In this study, CFT identified more positive goat sera than RBT or I-ELISA (Table 1). This is in agreement with many studies that have evaluated the sensitivity and specificity of CFT, showing a slightly to moderately better overall performance in general compared with RBT [18]. However, a more comprehensive study with more sera is needed to evaluate the performance of the serological test in Oman.

This study also highlighted some of challenges encountered during sampling. Some of farmers were not willing to allow us to take samples from their livestock because they are their source of living and also because of the misconception that sampling may harm them or they would be confiscated. Lack of cooperation was also encountered in some farms which caused difficulties in collecting blood samples.

Assessment of effectiveness of control measures taken by the MAF necessitates a comprehensive serological study but limitations beyond our control especially on part of village community owning livestock limited our access to wider sampling. Suffice it to say that this study paves the way for a detailed investigation on brucellosis diagnosis and control of the disease.

The most important finding in this study was the isolation, identification and molecular characterization of *Brucella melitensis* in a cow, which is not a preferential host for *Brucella melitensis*. A low seroprevalence in cattle suggests a spillover of *B. melitensis* from small ruminants to cattle in the herd.

A fundamental prerequisite for strategic planning of *Brucella* control is the identification of the species from infected animals. Akin to this is the analysis of procured infected material at molecular level and for an insight into the mode of transmission, both being crucial elements in epidemiological documentation. Relevant to this is the report from Italy and France on infection of *Brucella melitensis* in cattle where the source of *Brucella melitensis*was traced back to its preferential hosts, namely, small ruminants [17, 19].

## Conclusions

The present study reveals the serological profile of farm animals (sheep, goats, camel and cattle) in Saham before, during and after the human brucellosis outbreak and subsequent control measures by the Ministry of Agriculture and Fisheries. The retrospective and prospective patterns of seroreactivity in animals’ serum analysis reflects a clear decline in brucellosis prevalence in Saham area. Therefore, it can be concluded that the implemented strategy was successful in achieving its goal in controlling *Brucella* infection in Saham. However, limited biosecurity measures may have limited the long term effect of the program.

The successful isolation and biochemical identification of *Brucella melitensis* and its confirmation by Multiplex PCR supports serological tests and indicates that *Brucella melitensis* is most likely the causative agent of the disease in the area.

Therefore, based on the above information, the following recommendations need to be considered:

- Strict enforcement of law and policy for importation of animals and quarantine process regulations need to be implemented by the authorities.
- Increasing farmer’s knowledge and raise their awareness about the transmission of brucellosis and the zoonotic risks associated with it for better future control of the disease in the study area.

## Methods

### Study areas

**Fig. 2:**
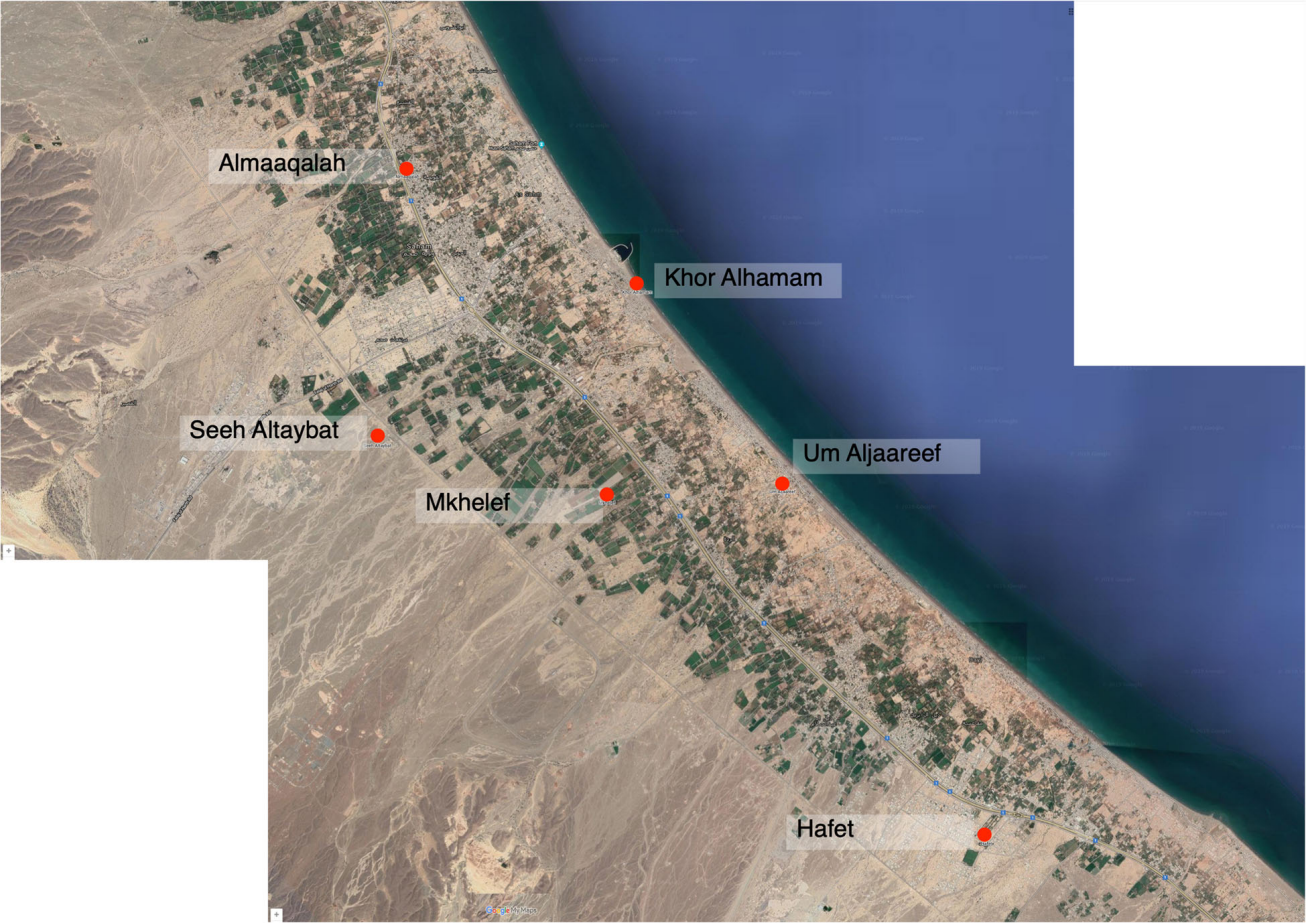
Composite image GoogleMap satellite view of the sampling site

The present study was conducted in Saham city, at the coastal Region of Al Batinah, northeast of Oman (Fig. 2). The city is located at 24.17 latitude and 56.89 longitudes at an elevation 2 m above sea level and has a population of 89,327 making it the third biggest city in Al Batinah.

### Sample size in the study area

This study was conducted in animals between 1 and 5 years of age. The health status, age, sex, and history of abortion for each animal were documented. There was no previous history of vaccination of animals in Saham. Regarding management, goats, sheep and cattle were kept under a mixed management system but camels were kept separately. Collection of sera was conducted in three different phases with 6 months period between each phase as follows:

The **Phase 1** study was conducted 6 months prior to the human brucellosis outbreak with the main aim of identifying infected animals guided by the local veterinarians, based upon samples already collected. The sera in this phase included 32 goats, (twelve males and twenty females), 38 sheep (fifteen males and twenty three females). One cow and seventeen camels (ten females and seven males) were also sampled.

The **Phase 2** study was conducted by MAF following the human brucellosis outbreak and covered the entire animal population in Saham. No details of gender or age were revealed to the team from MAF.

The **Phase 3** study was planned and conducted by our research group 6 months after the human brucellosis outbreak. This study was carried out in six randomly selected villages (five holdings) within the area where MAF carried out the brucellosis survey.

In phase 3, we sampled approximately 100 animals of each species, (400 in total). A sample size of 100 is sufficient to detect at least one infected animals with 95% confidence if the prevalence is above 3% (AusVet Epidemiological calculator), given a perfect sensitivity of the method. We did not adjust for test properties in this study.

### Ethical consideration

Before blood sample collection, permission was granted by the Ministry of Agriculture and Fisheries to access the farms in the study area. Collection of blood samples was carried out by professional veterinary technologists adhering to the regulations and guidelines on animal husbandry. The study was not an experimental study on animals and therefore approval by the ethical committee at Sultan Qaboos University was not needed. The study did not involve endangered or protected species.

In each village, a meeting was held with the community members to explain the purpose of the study. Farmers were not forced to participate in the survey, nor donate blood from their animals. Name, region and village of the farmers were registered.

### Blood sample collection

Five ml blood was collected from the jugular vein into Vacutainer tubes without anticoagulant for separation of the serum (EUROMED International, European Union) and Ethylenediaminetetraacetic acid (EDTA) tubes (EUROMED International, European Union) for bacterial culture and DNA extraction.

The samples were carefully collected and packed, avoiding any possibility of leakage or cross-contamination. All samples, blood, milk, or organs were packed in a cool box with ice packs and maintained cool during transport from the site of collection to the laboratory.

For serum separation, the blood tubes were centrifuged at 1370 x g for 5 min then the sera were kept at − 20 °C until further testing.

### Serological tests

#### Rose Bengal test (RBT)

All sera were tested using the Rose Bengal (RBT) according to the procedures described by the OIE [6]. For phase 2, the RBT antigen was from (Ellie LLC, Wisconsin, USA). For phase 1 & 3, a commercial RBT antigen (IDEXX batch 392–10) was used standardizing against OIE guide lines. For the test, the RBT antigen, test and control sera were brought to room temperature 30 min before use as homogeneous suspensions. 25 μl of serum and of antigen were adjacent to each other on agglutination plate consisting of 48 white tiles; mixed together thoroughly and rapidly and the plate was shaken lightly for 4 min. The degree of agglutination reactions was recorded immediately under good light and with the naked eye with a cut off time of 4 min, after which agglutinates revealed were not taken into consideration.

#### Indirect enzyme-linked immunoabsorbent assay (I-ELISA)

For sheep and goats, an IDEXX brucellosis Ovine/Caprine kit (IDEXX batch 4067) was used according to the manufacturer’s recommendations. Briefly, 190 μl of the dilution buffer were added to each well used for test sera and controls. Then, 10 μl of the controls and 10 μl of sera samples were added to the dilution buffer and homogenized gently by shaking the plates. The plates were covered with an adhesive cover and incubated for 45 min (± 5) at 18–26 °C. After incubation, the plates were washed three times using 300 μl wash buffer followed by complete drying. 100 μl of conjugate diluted 1:100 in conjugate buffer were dispensed in each well. Then the plates were covered with an adhesive cover and incubated for 30 min (± 5) at 18–26 °C. After the second incubation, the washing process was repeated, followed by addition of 100 μl TMB substrate solution into each well. The plates were incubated in a dark place for 10 min at 18–26 °C. 100 μl of the stop solution were added into each well to stop the reaction with gentle shaking by tapping the plates. The optical density (OD) was measured at 450 nm using an ELISA reader. Serum samples with a sample positive (S/P) ratio of 3.0 and greater were considered positive. Cattle sera were tested as described above except that *Brucella abortus* Antiboday Test kit (IDEXX) was used.

#### Competitive enzyme-linked immunoabsorbent assay (c-ELISA)

Camel’s sera were tested using an Ellie *Brucella* Antibody Test Kit (Ellie LLC, Wisconsin, USA) according to the instructions of the manufactures. Briefly, 50 μl of the samples were added to each test well. Then, an amount of 50 μl of controls was dispensed in duplicate test wells. A diluent of 1:100 of competing antibody which is stored at − 20 °C was prepared, and then 50 μl of it was added into all test wells. After that the plates were covered with a protective foil and incubated for 1 h at 37 °C. The plates were washed four times with 300 μl per of the wash then dried completely by tapping the plates firmly on an absorbent paper. Then 100 μl of conjugate (1:100) was dispensed to all wells. The plates were incubated uncovered for 30 min at room temperature. The washing process was repeated. 100 μl of substrate was then dispensed to all wells followed by incubating the plates for 15 min (± 3 min) at room temperature. Finally, a stop solution of 2 M H_2_SO_4_ (100 μl) was added to each well and optical density (OD) was measured at 450 nm using an ELISA reader.

#### Complement fixation test (CFT)

The CFT test was carried out in the brucellosis reference laboratory in Paris, France, according to the World Organization for Animal Health prescribed procedure as previously described [6, 18]. All reagents of CFT were obtained commercially (IDEXX batch 79, France). The antigen was a suspension of *Brucella abortus* (Weybridge 99 strain) inactivated by heat and phenol. The assay was carried out in a microtitre format by cold fixation with two units of complement. Test sera, positive and negative sera were diluted twofold in Veronal Buffer (VB) starting from 1:5 dilutions. Diluted serum samples (1:5) were inactivated for 30 min at the optimum temperature recommended by OIE guidelines for ruminants for each species. The minimum hemolytic dose (MHD) was estimated for each test set-up using 2% sensitized sheep red blood cell (SRBC) in VB. Two MHD units were used throughout the test. The end point titer was taken as the first well showing approximately 50% lysis of SRBC. Serum dilutions of 1:5 or higher giving a titer equivalent to 20 international CFT units (IU)/ml or more were considered as positive for the CFT. The results were expressed according to the percentage of the observed hemolysis. Titration was expressed in international units of complement fixation per milliliter (IU/ml). According to OIE and EU requirements, a result of 20 IU/ml (at least 50% of haemolysis inhibition at ¼ dilutions) is positive.

### Bacteriological examination

A serologically *Brucella* positive cow was slaughtered at Saham Slaughter house. Samples were collected from the lymph nodes (retropharyngeal, prefemoral, mandibular, mesenteric, external and internal inguinal, medial iliac, prescapular, parotid, retropharyngeal, bronchial, mediastinal, and mammary), udder, uterus and spleen. The specimens were collected aseptically, placed in sterile bags in a cool box with ice packs and transported to the laboratory.

A total of 25 samples were bacteriologically tested. Several grams of tissue (testis, uterus, or lymph node) were homogenized in sterile PBS and inoculated on blood agar, *Brucella* agar (*Brucella* medium base, Oxoid CM0169, Oxoid Ltd., Basingstoke, UK), and Farrell’s selective growth medium. Plates were incubated at 37 °C under normal atmospheric conditions and also with the addition of 10% CO_2_. Colony growth was checked daily and was usually observed after 3–5 days. Colonies were identified based on colonial morphology, Gram staining and biochemical tests. Further identification was performed using a Vitek 2 system (version 07. 01, BioMeriux).

### *Brucella* genomic DNA extraction from bacterial isolates

DNA isolation kit (Qiagen, Germany) was utilized for extracting DNA from *Brucella* isolated from the bronchial lymph node. A few colonies were directly mixed with 200 μL PBS in a 1.5 ml microcentrifuge and homogenized by vortexing. After adding a volume of 20 μL proteinase K, the contents of each tube was incubated for 5 min with μl RNaseA (100 mg/ml) followed by the addition of 200 μl lysis buffer (AL) and vigorous shaking up and down for 15 s while the tubes were perfectly sealed before centrifugation at 5867 x g for 1 min. After adding a volume of 200 μl ethanol (96–100%), the tubes were again shaken up and down vigorously for 15 s, and the the mixture (maximum 900 μl) was carefully transferred to the DNeasy columns. To facilitate elution of the lysate through the DNeasy columns, the tubes were centrifuged at 5867 x g for 1 min. For removal of residual contaminants, each of the samples was treated with 500 μl of wash buffer AW1 with centrifugation for 1 min at 5867 x g. This was followed by an additional wash with 500 μl of wash buffer AW2 and centrifugation at 17,968 x g for 3 min. For DNA elution, each of the tubes was incubated with a volume of 100 μl AE elution buffer at room temperature (15–25 °C) for 5 min for 5 min. DNA was eluted by centrifugation for 1 min at 5867 x g and the yielded volume of DNA was collected and stored at − 20 °C until analyzed.

### Molecular detection (multiplex PCR)

This procedure was carried out using INgene Bruce ladder (INgene Bruce ladder V®: Batch No 180515, Ingenasa, Madrid, Spain). The kit included five primer pairs (Table 5) designed on the strain-specific genetic differences. This technique was used for molecular typing of different *Brucella* at the species level [20]. DNA was amplified by PCR (Veriti 96 well, Applied Biosystem, USA) where cycling was initiated by denaturing at 95 °C temperature for 4 min, then 35 cycles at 94 °C for 45 s, 45 s at 60 °C, 60 s at 72 °C then 72 °C for 7 min for final extension. An ethidium bromide (0.5 μg/mL)-stained 1% agarose gel was used for analysis of PCR fragments. The gels’ results obtained by UV illumination were saved in a documentation and analysis system. Identification of *Brucella* species was based on the corresponding molecular size of amplified fragments in a parallel DNA ladder (100 bp and 1Kb).

**Table 5:** Primer sets for Bruce ladder multiplex PCR

Primer	Sequence (5′–3′)	Amplicon size (bp)
BMEI0998f BMEI0997r	ATC-CTA-TTG-CCC-CGA-TAA-GG GCT-TCG-CAT-TTT-CAC-TGT-AGC	1682
BMEII0843f BMEII0844r	TTT-ACA-CAG-GCA-ATC-CAG-CA GCG-TCC-AGT-TGT-TGT-TGA-TG	1071
BMEII0428f BMEII0428r	GCC-GCT-ATT-ATG-TGG-ACT-GG AAT-GAC-TTC-ACG-GTC-GTT-CG	587
BR0953f BR0953r	GGA-ACA-CTA-CGC-CAC-CTT-GT GAT-GGA-GCA-AAC-GCT-GAA-G	272
BMEI0752f BMEI0752r	CAG-GCA-AAC-CCT-CAG-AAG-C GAT-GTG-GTA-ACG-CAC-ACC-AA	218

## Data Availability

The datasets used and/or analyzed during the current study are available from the corresponding author on reasonable request.

## Abbreviations

C-ELISA: Competitive enzyme linked immunosorbent assay
I-ELISA: Indirect enzyme linked immunosorbent assay
IU: International unit
MAF: Ministry of Agriculture & Fisheries
MHD: The minimum hemolytic dose
Min: Minutes
OD: The optical density
RBT: Rose Bengal test
S/P: Sample positive
SRBC: Sensitized sheep red blood cell
TMB: 3,3′,5,5′-Tetramethylbenzidine
VB: Veronal Buffer
