# Correction to: Investigation on Brucella infection in farm animals in Saham, Sultanate of Oman with reference to human brucellosis outbreak

**DOI:** 10.1186/s12917-020-02316-4

**Published:** 2020-03-24

**Authors:** Yasmin ElTahir, Anfal Al-Farsi, Waleed Al-Marzooqi, Alghalya Al-Toobi, Osman M. Gaafar, Maryne Jay, Yannick Corde, Shekar Bose, Abeer Al-Hamrashdi, Kaadhia Al-Kharousi, Sunil Rajamony, Muhammed Nadeem Asi, Nasseb Al-Saqri, Rudaina AlBusaidi, Elshafie I. Elshafie, Eugene H. Johnson

**Affiliations:** 1 Department of Animal & Veterinary Sciences, College of Agricultural & Marine Sciences, Sultan Qaboos University, P.O.box 34, 123 Alkhod, Sultanate of Oman; 2 Paris-Est University/Anses, EU/OIE/FAO & National Reference Laboratory for Brucellosis, Animal Health Laboratory, Maisons-Alfort, France; 3 Department of Natural Resources Economics, Sultan Qaboos University, P.O.box 34, 123 Alkhod, Sultanate of Oman

**Correction to: BMC Vet Res (2019) 15:378**

**https://doi.org/10.1186/s12917-019-2093-4**

The original article [1] incorrectly presents final author, Eugene H. Johnson’s name incorrectly whereby middle initial, ‘H.’ is mistakenly presented as a Family Name.
